# DMX-200 in Patients With Primary Focal Segmental Glomerulosclerosis: Results of the Phase 2 ACTION2 Trial

**DOI:** 10.1016/j.ekir.2025.09.044

**Published:** 2025-10-10

**Authors:** Hiddo J.L. Heerspink, Alessia Fornoni, Lesley A. Inker, Irene L. Noronha, Hernán Trimarchi, Muh Geot Wong, Alisha Smith, Robert Shepherd, Carl W. White, David Fuller, Jonathan Barratt

**Affiliations:** 1 Department of Clinical Pharmacy and Pharmacology, University of Groningen, University Medical Center Groningen, Groningen, The Netherlands; 2 Katz Family Division of Nephrology and Hypertension, Department of Medicine, University of Miami, Miller School of Medicine, Miami, Florida, USA; 3 Division of Nephrology, Department of Medicine, Tufts Medical Center, Boston, Massachusetts, USA; 4 Laboratory of Cellular, Genetic, and Molecular Nephrology, Division of Nephrology, University of São Paulo School of Medicine, São Paulo, Brazil; 5 Nephrology Service, Hospital Británico de Buenos Aires, Buenos Aires, Argentina; 6 Faculty of Medicine and Health, University of Sydney, Sydney, New South Wales, Australia; 7 Concord Repatriation General Hospital, Sydney, New South Wales, Australia; 8 Dimerix Bioscience, Fitzroy, Victoria, Australia; 9 University of Leicester, Leicester, UK

**Keywords:** ARB, AT1R, CCR2, DMX-200, FSGS, repagermanium

## Introduction

Focal segmental glomerulosclerosis (FSGS) is a leading cause of nephrotic syndrome and proteinuria in adults and children, with a reported incidence of 0.2 to 2.5 of 100,000/yr.^1^ The etiology, natural history, and response to treatment of FSGS is heterogeneous arising from multiple idiopathic, genetic, and external drivers of podocyte damage.^2^ FSGS is associated with significant morbidity and mortality accounting for 3% of adult and 10% to 15% of pediatric kidney failure. Typically, 50% of patients require renal replacement therapy within 10 years of diagnosis.^3^

Patients with FSGS are routinely treated with angiotensin-converting enzyme inhibitors or angiotensin receptor blockers (ARBs) often in combination with corticosteroids and immunosuppressive drugs. The treatment goal is to reduce proteinuria as one of the strongest predictors of kidney survival in patients with FSGS.^4^ However, remission of proteinuria is often not achieved with currently available treatments and immunosuppressive agents are associated with significant side effects.^2^ Thus, there remains a critical unmet need for well-tolerated treatments that effectively reduce proteinuria in this population.

C-C chemokine receptor 2 (CCR2) inhibitors are promising agents for the treatment of FSGS. Glomerular expression of CCR2 and its ligand chemokine (C-C motif) ligand 2 are significantly increased in patients with FSGS compared with healthy controls,^5^ and may contribute to glomerular and interstitial scarring through an increase in macrophage recruitment. Activation of CCR2 podocyte expression results in increased cellular motility, rearrangement of the actin cytoskeleton, and increased permeability to albumin.^6^ Preclinical studies indicate that pharmacological inhibition of CCR2 or CCR2 genetic knockout both decrease proteinuria and reduce glomerular injury.^5^^,^^7^ Furthermore, both CCR2 and angiotensin 2 receptor type 1 are expressed on podocytes and other kidney cells and may form functional complexes that enhance receptor signalling.^7^

DMX-200 (repagermanium) is a CCR2 pathway inhibitor which reduces monocyte chemotaxis *in vitro*^8^ and ameliorates markers of inflammation and fibrosis in animal models of renal disease.^9^ In the subtotal nephrectomy model of progressive kidney injury, the combination of an ARB and DMX-200 synergistically blocks proteinuria and kidney macrophage infiltration while preserving podocytes.^7^ We hypothesized that simultaneous inhibition of CCR2 and angiotensin 2 receptor type 1 would inhibit CCR2-mediated inflammatory pathways on podocytes and monocytes and reduce proteinuria in patients with FSGS. We initiated a small proof of concept clinical trial to test this hypothesis.

## Results

### Patient and Demographic Characteristics

**Figure 1:**
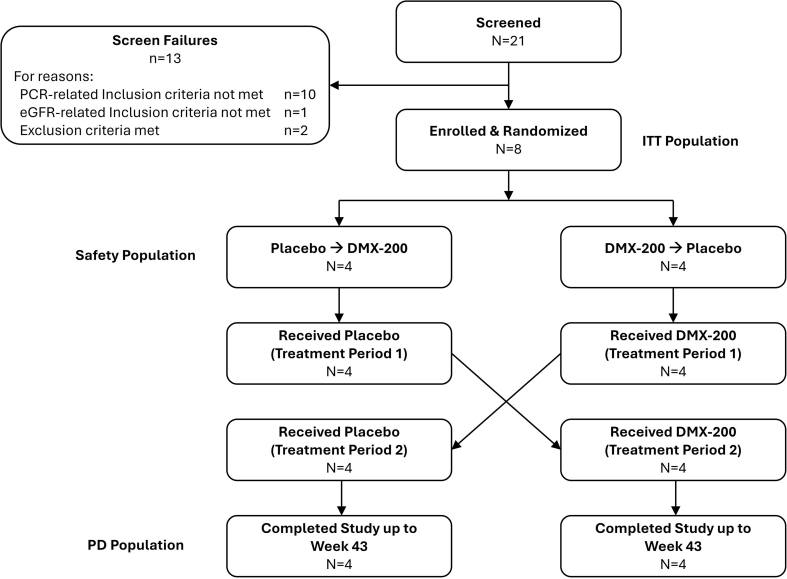
Disposition of patients in the study. eGFR, estimated glomerular filtration rate; ITT, intent-to-treat; PCR, urinary protein-to-creatinine ratio; PD, pharmacodynamic.

A randomized crossover clinical trial was designed (Supplementary Methods and Supplementary Tables S1 and S2). Eight participants (4 in each group) were assigned to 16-week treatment periods of DMX-200 or placebo in random order separated by a 6-week wash-out period (Figure 1 and Supplementary Figure S1). Baseline demographic characteristics were similar between treatment groups and treatment period (Table 1 and Supplementary Table S3). Screen failure (Figure 1) and treatment compliance are reported in the Supplementary Results.

**Table 1:** Baseline demographics and characteristics

Variable (unit) category	Placebo→DMX-200 (*n* = 4)	DMX-200→Placebo (*n* = 4)
Age (yrs), mean (SD)	47.3 (13.1)	44.5 (17.1)
Sex		
Male	2 (50.0%)	3 (75.0%)
Female	2 (50.0%)	1 (25.0%)
Race		
White	3 (75.0%)	3 (75.0%)
Other	1 (25.0%)	1 (25.0%)
BMI at screening (kg/m^2^), mean (SD)	27.0 (5.4)	29.6 (6.7)
Blood pressure (mm Hg), mean (SD)		
Systolic	131.5 (11.3)	134.8 (10.4)
Diastolic	78.8 (5.7)	79.8 (5.4)
eGFR (ml/min per 1.73 m^2^), mean (SD)	77 (35.3)	33.4 (15.7)
UPCR (mg/mmol), median (25th & 75th percentiles)	205.1 (202.6–214.9)	374.6 (326.9–537.9)
ARB use (irbesartan 300 mg)	4 (100%)	4 (100%)
Concomitant use of diuretic, (%)	3 (75%)	2 (50%)
Concomitant use of calcineurin inhibitor, (%)	0 (0%)	2 (50%)
Concomitant use of steroids (%)	0 (0%)	2 (50%)

ARB, angiotensin receptor blocker; BMI, body mass index; DMX-200, repagermanium; eGFR, estimated glomerular filtration rate; *n*, number of patients in each category; UPCR, urinary protein-to-creatinine ratio; %, percentage of patients in each category calculated relative to the total number of patients in the relevant population.

Age (yrs) is relative to the date the informed consent form was signed.

### Safety and Tolerability

DMX-200 was generally very well-tolerated. The frequency of adverse events (AEs) was similar in the placebo and DMX-200 treatment groups with 6 (75%) and 7 participants (87.5%), respectively reporting AEs. The reported AEs were consistent with the age and comorbidities of the participant population with no clinically relevant findings in laboratory parameters, vital signs, or electrocardiogram results. There were no discontinuations because of treatment-emergent AEs. A serious AE (tendonitis) was reported but was considered unrelated to study treatment. Most AEs were mild, with 4 moderate AEs. All treatment-emergent AEs are reported in Supplementary Table S4.

### Efficacy

As an exploratory pilot study, *P*-values for efficacy were not generated; 95% confidence intervals (CIs) are provided to facilitate results interpretation. The per-protocol analysis of antiproteinuric efficacy assessed the geometric mean ratio of the log-transformed urine protein-to-creatinine ratio comparing baseline to end of treatment, using a random effects mixed model. The results were 0.80 (*n* = 8, 95% CI; 0.59–1.07), reflecting a 20% reduction in proteinuria following treatment with DMX-200, and 0.91 (*n* = 8, 95% CI; 0.67–1.22) reflecting a 9% reduction in proteinuria following treatment with placebo, corresponding to a between-group difference of 12% (geometric mean ratio: 0.88 [95% CI: 0.63–1.22]). Additional prespecified sensitivity analyses, *post hoc* analysis of urine protein-to-creatinine ratio as well as urine protein-to-creatinine ratio per treatment period and group (Supplementary Table S5), urine albumin-to-creatinine ratio (Supplementary Table S6), and estimated glomerular filtration rate are reported in the Supplementary Results. Although trends to lower proteinuria were observed across all predefined subgroups, the intent-to-treat population showed no statistically significant results between placebo and DMX-200. Finally, an exploratory biomarker analysis of urine ligand chemokine (C-C motif) ligand 2 concentration between baseline and end of treatment observed a geometric mean ratio of DMX-200 or placebo treatment groups versus baseline of 0.83 (*n* = 8, 95% CI; 0.60–1.17) and 1.11 (*n* = 8, 95% CI; 0.79–1.56) respectively, corresponding to a placebo-adjusted relative change from baseline of 25% (geometric mean: 0.75 [95% CI; 0.47–1.21]).

## Discussion

Patients with FSGS who have proteinuria are at high risk of kidney failure and currently there are no approved therapies available. For patients presenting with nephrotic syndrome, high dose systemic corticosteroids are used as an empiric first line treatment, with the use of nonsteroidal immunosuppressants reserved for persistently nephrotic patients. Response to these agents varies considerably and given the high morbidity and typically rapid disease progression of FSGS, there is an urgent need for a proven effective, well-tolerated therapy that reduces proteinuria and prolongs kidney function.

The primary outcome of this study was safety and tolerability of DMX-200 in patients with FSGS, and the study was not powered to resolve secondary (efficacy), tertiary, or biomarker objectives. The small sample size, relatively short duration of therapy, inherent variability of the response, and the potential for residual treatment effect in the crossover design limits interpretation of efficacy, with further larger and longer studies required to establish both the magnitude DMX-200’s anti-proteinuric effect and the potential to slow glomerular filtration rate decline in patients with FSGS.

The combination of DMX-200 and an ARB has been very well-tolerated throughout clinical development. In 3 completed phase II studies (*n* = 80) including the results reported here, the incidence, nature, and severity of reported treatment-emergent AEs was similar in both DMX-200 and placebo groups. The emerging safety profile and tolerability of DMX-200 appears well-suited for chronic use.

Mechanistically, blockade of CCR2 by DMX-200 should reduce inflammation-mediated podocyte damage as well as podocyte injury caused by activation of CCR2 expressed on podocytes. Notably, in this study DMX-200 reduced the concentration of the urinary ligand chemokine (C-C motif) ligand 2 biomarker suggesting a reduction in inflammatory cell activation and recruitment to the kidney. However, because this small study was not statistically powered, these nonsignificant observations require further investigation. The use of DMX-200 in combination with an ARB is based on increased inhibition of CCR2 signaling in cell-based pharmacology studies, and *in vivo* studies in the subtotal nephrectomy rat model of FSGS, where the combination of DMX-200 and an ARB provided a greater reduction in proteinuria, macrophage infiltration, and podocyte loss than vehicle or either antagonist alone.^7^ The known roles of angiotensin 2 receptor type 1 and CCR2 individually in the progression of renal fibrosis, and the synergistic effect of simultaneous receptor inhibition combine to provide a strong therapeutic rationale for the combination approach used in this Phase II trial.

In conclusion, in this small, short duration, proof-of-concept study, DMX-200 combined with stable ARB treatment demonstrated good tolerability in patients with FSGS. Given the significant unmet medical need for new FSGS therapies, these data, in combination with the totality of available preclinical and clinical data for DMX-200, supported a larger study to investigate the long-term efficacy of DMX-200 in reducing proteinuria and estimated glomerular filtration rate decline in patients with FSGS (NCT05183646 / ACTION3).

## Disclosure

HJLH reports funding for clinical trials from AstraZeneca, Bayer, Boehringer Ingelheim, Janssen, and Novo Nordisk (all to the University of Groningen); consulting fees from AstraZeneca, Alexion, Alnylum, Bayer, Boehringer Ingelheim, Biocity Pharmaceutics, Dimerix, Eli Lilly, Gilead, Idorsia, Janssen, Novartis, Novo Nordisk, Roche, and Travere Therapeutics; and honoraria for lectures from AstraZeneca, Bayer, and Novo Nordisk. MGW has received fees for advisory boards, steering committee roles, or scientific presentations from Travere, Vertex, Baxter, Amgen, AbbVie, Chinook, Dimerix, Otsuka, GlaxoSmithKline, and CSL-Behring. LAI has received funding for research support to the Tufts Medical School from the NIH, NKF, Chinook, River 3 Renal, and Alexion. LAI has received funding for consulting, steering committee or DSMB committees to the Tufts Medical School from AstraZeneca and Alexion. AF is one of the inventors on pending patents (PCT/US2019/032215; US 17/057,247; PCT/US2019/041730; PCT/US2013/036484; US 17/259,883; US17/259,883; JP501309/2021, EU19834217.2; CN-201980060078.3; CA2,930,119; CA3,012,773; CA2,852,904) or issued patents (US10,183,038 and US10,052,345) aimed at preventing and treating renal disease; stands to gain royalties from their future commercialization; is vice president of L&F Health LLC; is a consultant for ZyVersa Therapeutics, Inc, which has licensed worldwide rights to develop and commercialize hydroxypropyl-beta-cyclodextrin for the treatment of kidney disease from L&F Research, which was partially funded by L&F Health LLC; and holds equities in Renal 3 River Corporation. AS, RS, CWW, and DF are employees of Dimerix. All the other authors declared no competing interests.

## Supplementary Material

Supplementary File (PDF) **Supplementary Methods. Supplementary Results. Figure S1.** Schematic of the study design. **Table S1.** Inclusion and exclusion criteria. **Table S2.** Study assessment schedule. **Table S3.** Baseline characteristics at start of placebo or DMX-200 treatment periods. **Table S4.** Adverse events in study population. **Table S5.** Analysis of 24-hour urine protein-to-creatinine ratio (UPCR) per treatment period and group. **Table S6.** Analysis of ratio of 24-hour urine albumin-to-creatinine ratio. CONSORT checklist.
