# Supplementary material for: DMX-200 in Patients With Primary Focal Segmental Glomerulosclerosis: Results of the Phase 2 ACTION2 Trial
Source: Kidney Int Rep. 2025 Oct 10;10(12):4272–6. doi: 10.1016/j.ekir.2025.09.044 (PMC12712502; doi:10.1016/j.ekir.2025.09.044)
Supplement: Supplementary File (PDF) — Supplementary Methods. Supplementary Results. Figure S1. Schematic of the study design. Table S1. Inclusion and exclusion criteria. Table S2. Study assessment schedule. Table S3. Baseline characteristics at start of placebo or DMX-200 treatment periods. Table S4. Adverse events in study population. Table S5. Analysis of 24-hour urine protein-to-creatinine ratio (UPCR) per treatment period and group. Table S6. Analysis of ratio of 24-hour urine albumin-to-creatinine ratio. CONSORT checklist. [file mmc1.pdf]

Supplementary Material

Supplementary Methods

The ACTION study has been reported on clinicaltrials.gov (trial registration: NCT03649152) and the study was conducted in accordance with international guidelines including the Declaration of Helsinki, applicable International Council for Harmonisation (ICH) Good Clinical Practice (GCP) Guidelines and the Australian Therapeutic Goods Administration (TGA) regulations. All approvals by local human research ethics committees were obtained prior to enrolment of patients at each site.

Study participants and sites

Male or female patients 18 to 80 years (inclusive) of age with biopsy-proven primary FSGS who had received a stable dose of 300 mg daily of irbesartan (in any marketed formulation) for at least 3 months prior to screening were eligible to participate in the study. Inclusion and exclusion criteria are listed in Supplementary Table S1. Patients were on study at eight sites between November 2018 and July 2020 in Australia.

Study design and treatment

A schematic of the study design is presented in Supplementary Figure S1. Investigators obtained informed consent from patients prior to entering the screening process. The study consisted of a 14-day screening period (including a screening visit and baseline assessment prior to randomisation), two 16-week treatment periods (treatment period 1 and treatment period 2) with a 6-week washout period between the treatment periods, followed by a 4-week follow-up period. The total study duration per patient was approximately 45 weeks. On day 1, eligible patients who met all study eligibility criteria during screening and baseline assessments were randomised via an online central randomisation module in a 1:1 ratio to either one of two treatment sequence groups:

- DMX-200 then placebo: DMX-200 (120 mg twice daily) in treatment period 1 followed by placebo twice daily in treatment period 2 or;

- Placebo then DMX-200: Placebo twice daily in treatment period 1 followed by DMX-200 (120 mg twice daily) in treatment period 2

The 120 mg BID dose of DMX-200 was selected based on pharmacometric modelling of the dose required to provide a target minimum mean blood concentration of DMX-200 of 120 ng/ml. Patients also continued to receive their ongoing daily dose of oral 300 mg irbesartan (in any marketed formulation) as prescribed per standard of care.

Endpoints and assessment

The primary endpoints of the study were incidence and severity of adverse events (AEs) and changes in clinical status and laboratory measures in patients treated with DMX-200 compared to placebo. Patients were evaluated by investigators for adverse events (AEs) at each protocol-scheduled study visit (Supplementary Table S2). Vital signs (including height, weight, BMI, body temperature, respiratory rate, pulse rate and systolic and diastolic blood pressure) were taken at each protocol-scheduled time point. Clinical safety laboratory data (haematology, biochemistry, and urine chemistry) were also taken at each protocol-scheduled time point and categorised according to reference ranges (low, normal, high). Treatment-emergent AEs were grouped by system organ class and preferred term and assessed for severity (mild, moderate or severe) and relationship to treatment (not related, unlikely, possible, probable, or highly probable). Patient treatment compliance and exposure with DMX-200 and the background therapy irbesartan was calculated based on the number of doses received compared to the number planned during the treatment period.

Secondary efficacy/pharmacodynamic endpoints included percent change from baseline in 24-hour UPCR after the end of therapy with DMX-200 compared to placebo as well as proportion of patients who achieved a response at any stage during treatment with DMX-200 as compared to placebo. For clarity 'the end of treatment' was defined as the mean of all assessments collected during the 15th and 16th week of treatment. For treatment period 1 (Supplementary Figure S1), the end of treatment was defined as the log-transformed mean urinary protein:creatinine ratio (UPCR) values collected at weeks 15 and 16 (visits 6 and 7 respectively, Supplementary Table S2). For treatment period 2 (Supplementary

Figure S1), the end of treatment was defined as the mean of the data at study weeks 38 and 39 (visits 12 and 13 respectively, Supplementary Table S2).

Exploratory endpoints included assessment of irbesartan and DMX-200 concentration in urine and plasma. Change from baseline after treatment with DMX-200 as compared to placebo in markers of renal function, i.e., urine albumin-to-creatinine ratio (UACR); urine albumin concentration; total urine albumin excretion; urine protein concentration; total urine protein excretion; serum creatinine; creatinine clearance; estimated glomerular filtration rate (eGFR, calculated using the chronic kidney disease epidemiology collaboration (CKD-EPI) formula) were also reported. Additional exploratory analysis was performed to determine change in 24-hour urine for CCL2 from baseline after treatment with DMX-200 as compared to placebo. Patient clinic visits and details of full assessments performed at each visit are outlined in Supplementary Table S2. Secondary and exploratory end points peripheral blood/serum and urine samples were analyzed at a central laboratory.

Statistical Analysis and Data Presentation

As this study had a crossover design, 2 types of study baselines were defined, 1) the study baseline and 2) the baselines for the 2 separate treatment periods (see Supplementary Table S2). Data collected at the study baseline (prior to week 0) included assessments collected during the screening (week −2) and baseline 1 (week −1) visits: Demographics, baseline disease characteristics, medical history, and prior medications. The baseline for treatment period 1 was the same as the study baseline and included means of the data collected during the screening and baseline 1 visits (weeks −2 and −1). The baseline for treatment period 2 included means of the data collected at the baseline 2 visits during weeks 22 and 23.

As the primary outcome of the study was safety and tolerability of DMX-200 in patients with FSGS, the study was not designed to be powered to resolve secondary or tertiary objectives. For secondary efficacy assessment, we used a pre-specified random effects mixed model on the log transformed UPCR, with patient nested in sequence included as a random effect and treatment, period, and sequence included as fixed effects. Log transformation for the data was used due to non-normal distribution of

UPCR. p-values are not reported due to the exploratory nature of the secondary endpoint analysis. Data from the random effects mixed model are reported as geometric means of UPCR ratio and the 95% CI based on the random effects mixed model by exponentiating the estimated least squares means and their CIs. The treatment effect is reported as a placebo corrected ratio (DMX-200 versus placebo) using the same model as the ratio of geometric means of DMX-200 versus placebo by exponentiating the difference of estimated least squares means and its CIs. A pre-specified per protocol sensitivity analysis that only included UPCR data from patients with treatment compliance of ≥80% to both DMX-200, placebo and irbesartan and patients who did not change drugs that could impact proteinuria levels (e.g., diuretics, Vitamin D analogues, non-steroidal anti-inflammatory drugs, mineralocorticoid receptor antagonists, and corticosteroids) was performed.

All 8 patients (100%) completed the study up to the end of the follow-up period (up to Week 43) and there were no withdrawals from the study. There was no imputation for missing data.

Statistical analysis of the exploratory endpoints to determine change in 24-hour urine for CCL2 from baseline was not predefined and thus was exploratory in nature and performed post-hoc. Change from baseline at end of therapy for placebo and DMX-200 were calculated and expressed a percentage of the baseline measurement. A placebo corrected (DMX-200 minus placebo) treatment effect was also calculated.

Supplementary Results

Screen failure and treatment compliance

The most common reason for screen failure was a failure was screening or baseline urine proteinuria/creatine ratio (UPCR) values that were <150 mg/mmol, or UPCR values exceeding ±30% of the screening value at the baseline assessment. Compliance with investigation treatment was high (>90% prescribed doses taken) in both groups as was compliance to baseline irbesartan treatment (>95% prescribed doses taken).

Efficacy

Effect of DMX-200 on urine protein/creatinine ratio (UPCR)

In a pre-specified per protocol sensitivity analysis, the geometric mean of the log transformed UCPR versus baseline was 0.76 (n=3, 95% CI; 0.07 to 7.70) and 0.99 (n=3, 95% CI; 0.1 to 10.10) following treatment with DMX-200 or placebo respectively corresponding to a between group difference of 24% (geometric mean ratio 0.76 [95% CI 0.03, 20.29] in the Per Protocol population. Following database lock, poor treatment compliance (60%) was noted for a single patient. A post-hoc sensitivity analysis excluding this patient showed a geometric mean of the log transformed UPCR versus baseline of 0.78 (n=7, 95% CI; 0.54, 1.12) and 0.94 (n=7, 95% CI; 0.66, 1.35) following treatment with DMX-200 or placebo respectively corresponding to a between group difference of 17% (geometric mean 0.83 [95% CI; 0.57, 1.20]). Due to limitations of the mixed effects model for a study with limited patient numbers (n=8), a descriptive post-hoc analysis of non-log transformed UPCR per treatment period and group is also reported in Supplementary Table S5. This analysis was performed post-hoc and are of an exploratory nature. No statistical analysis was therefore performed. Similar results were observed for the treatment effect of DMX-200 on the urine albumin/creatinine ratio (ACR) compared to placebo (Supplementary Table S6).

Effect of DMX-200 on estimated glomerular filtration rate (eGFR)

No notable changes in exploratory measures of eGFR from baseline at end of therapy were observed, in addition, no substantial treatment group differences were observed indicating a lack of an acute change in eGFR due to haemodynamic changes.

Supplementary Table S1: Inclusion and exclusion criteria

Inclusion criteria

- Aged 18 to 80 years (inclusive) at Screening.
- A diagnosis of primary FSGS confirmed by renal biopsy.
- Must had received a stable dose of 300 mg daily of irbesartan (in any marketed formulation) for at least 3 months prior to Screening and had no plan to change treatment regimen throughout the study.
- Patients could be on stable doses of ACE inhibitors, aldosterone inhibitors, direct renin inhibitors and/or sodium-glucose co-transporter-2 (SGLT2) inhibitors. However, the dose and regimen were to be stable for 3 months prior to Screening and patients must have had no plan to change treatment regimen throughout the study.
- If patients were taking immunosuppressive medications (except for rituximab or cyclophosphamide), they must have had a stable treatment regimen for 3 months prior to Screening and were not to have plans to alter the regimen except to maintain therapeutic immunosuppression or in the event of an adverse event. Patients who received rituximab or cyclophosphamide must have ceased treatment for at least 6 months prior to Screening.
- Mean of 2 PCR values (Screening and Baseline) was $\geqslant$150 mg/mmol (1326 mg/mg), and within $\pm$ 30% of the Screening value at the Baseline assessment.
- Estimated glomerular filtration rate was $\geqslant$25 mL/min/1.73 m^2 using the Chronic Kidney Disease Epidemiology Collaboration formula at Screening.
- Serum potassium levels (Screening and Baseline) were $<$5.5 mmol/L. If either value was 5.5 mmol/L or above, the patient could receive dietary advice and be re-tested 1 week later.
- A female patient was eligible to participate if she was not pregnant, not breastfeeding, and at least 1 of the following conditions applied:
    - Not of childbearing potential, defined as surgically sterile or postmenopausal
    - Of childbearing potential and agreed to use a highly effective method of contraception consistently during the treatment period and for at least 60 days after the last dose.
- A male patient with a female partner of childbearing potential was eligible to participate if he agreed to use acceptable contraception (during the treatment period and for at least 60 days after the last dose and refrained from donating sperm during this period.
- Had given written informed consent prior to any study procedures being performed.

Exclusion Criteria

1. Had FSGS secondary to another condition.
2. A history of type 1 diabetes mellitus, diagnosis of type 2 diabetes mellitus prior to FSGS positive renal biopsy, or non-fasting blood glucose $>$10 mmol/L at Screening.

3. A prior organ or stem cell transplant.
4. A major adverse cardiac event within 6 months before Screening.
5. Lymphoma, leukaemia, or any malignancy within the past 5 years, except for basal cell or squamous cell carcinomas of the skin or cervical carcinoma in situ that were resected with no evidence of metastatic disease for 3 years.
6. Jaundice, active hepatitis, or known hepatobiliary disease (except asymptomatic cholelithiasis).
7. Alanine aminotransferase and/or aspartate aminotransferase >2 times the upper limit of normal at Screening.
8. Participation in any clinical study with an experimental medication or device within 90 days or 5 half-lives (whichever was longer) of Screening or had previously participated in a study involving PPG.
9. Positive Screening assessment for viral hepatitis B surface antigen (HBsAg) or hepatitis C virus (HCV) antibody AND positive HCV RNA or human immunodeficiency virus (HIV), or a history of illicit drug injection.
10. Seated blood pressure of ≥160/100 mm Hg at Screening.
11. Body mass index (BMI) ≥35 kg/m2 at Screening.
12. Past hospitalisation for a major depressive episode.
13. Was breast feeding or pregnant.
14. Was unable to comply with the study procedures and assessments, including the ability swallow capsules.
15. Any other disease, physical or psychological condition that the investigator or sponsor believed could contraindicate the use of the investigational medicinal product or affect the interpretation of study results or render the patient at high risk from treatment complications.
16. Were investigator site personnel directly affiliated with this study and their immediate families. Immediate family was defined as a spouse, parent, child or sibling, whether biological or legally adopted.

Supplementary Table S2: Study assessment schedule

		**STUDY PERIOD 1**							**Wash out**	**STUDY PERIOD 2**						**Follow-up**
		**Screen/B1**	**B1**	**Treatment Period 1**						**B2**	**B2/TP2**	**Treatment Period 2**				
**Week (Day) (+/- 3 days)**		**-2 (-14)**	**-1 (-7)**	**0 (1)**	**1 (8)**	**8 (57)**	**15 (106)**	**16 (113)**	***16-22***	**22 (155)**	**23 (162)**	**24 (169)**	**31 (218)**	**38 (267)**	**39 (274)**	**43 (302)**
**Visit number**	**Consent**	**1**	**2**	**3**	**4**	**5**	**6**	**7**	**-**	**8**	**9**	**10**	**11**	**12**	**13**	**14**
Visit to site		X	X	X	X	X	X	X		X	X	X	X	X	X	X
Informed consent[a]	X															
Eligibility assess/confirmation		X	X													
Demographics and medical history		X														
Prior medication history		X														
Height and weight[b]		X			X			X		X		X			X	X
Physical examination		X						X		X					X	X
Vital signs		X	X	X	X	X	X	X		X	X	X	X	X	X	X
12-Lead electrocardiogram		X						X		X					X	X
Serology		X														
Pregnancy/Postmenopausal Confirmatory screen[c]		X														
Adverse event reporting			X	X	X	X	X	X		X	X	X	X	X	X	X
Concomitant medications			X	X	X	X	X	X		X	X	X	X	X	X	X
Dispense diary cards		X														
Review of diary cards[d]			X	X	X	X	X	X		X	X	X	X	X	X	X
Randomisation				X												
IP dispensed				X		X					X		X			
IP taken[e]				X[f]	X[f]	X	X	X			X[f]	X[f]	X	X	X	
IP review					X	X	X	X				X	X	X	X	
**24-Hour Urine Collection**		X[g]	X			X	X	X		X	X		X	X	X	X
-Urinalysis		X	X			X	X	X		X	X		X	X	X	X
-Urine Chemistry		X[h]	X[h]			X	X	X		X	X		X	X	X	X

Week (Day) (+/- 3 days)	Consent	STUDY PERIOD 1							Wash out	STUDY PERIOD 2						Follow-up
		Screen/B1	B1	Treatment Period 1						B2	B2/TP2	Treatment Period 2				
**Week (Day) (+/- 3 days)**		**-2 (-14)**	**-1 (-7)**	**0 (1)**	**1 (8)**	**8 (57)**	**15 (106)**	**16 (113)**	*16-22*	**22 (155)**	**23 (162)**	**24 (169)**	**31 (218)**	**38 (267)**	**39 (274)**	**43 (302)**
**Visit number**	**Consent**	**1**	**2**	**3**	**4**	**5**	**6**	**7**	**-**	**8**	**9**	**10**	**11**	**12**	**13**	**14**
-Repagermanium concentration[i]			X			X	X	X		X	X		X	X	X	X
-Irbesartan concentration[i]			X			X	X	X		X	X		X	X	X	X
-CCL2 concentration[i]		X	X			X	X	X		X	X		X	X	X	X
-Inflammatory biomarker panel [i,j]			X					X			X				X	X
**Blood Sample Collection**		X	X		X	X	X	X		X	X	X	X	X	X	X
-Haematology		X	X		X	X	X	X		X	X	X	X	X	X	X
-Biochemistry		X	X		X	X	X	X		X	X	X	X	X	X	X
-Repagermanium concentration[i]			X		X	X	X	X		X	X	X	X	X	X	X
-Irbesartan concentration[i]			X		X	X	X	X		X	X	X	X	X	X	X
-ProMarkerD[i]			X		X	X	X	X		X	X	X	X	X	X	X
-CCL2 concentration[i]			X		X	X	X	X		X	X	X	X	X	X	X

Abbreviations: B1 = Baseline assessment for Period 1; B2 = Baseline assessment for Period 2; CTGF = connective tissue growth factor; IL = interleukin; IP = investigational product; NGAL = neutrophil gelatinase-associated lipocalin; MCP= monocyte chemoattractant protein; PCR = protein/creatinine ratio; TGF-β = transforming growth factor-β; TP = Treatment Period.

a. Informed consent was obtained prior to performing any study procedures. This was obtained within 4 weeks of Screening (for patients who were on stable irbesartan 300 mg/day for 3 months prior to entering the study) or prior to transitioning to irbesartan 300 mg/day for 3 months (if patients were on other anti-hypertensive regimens prior to Screening).
b. Height was recorded at the Screening visit only.
c. Female patients of childbearing potential were required to undergo a pregnancy screen (β-human chorionic gonadotropin, collected as part of biochemistry sample). Postmenopausal women had their FSH levels assessed to confirm postmenopausal status.
d. Patients were provided with diary cards to capture AEs, concomitant medications, IP and irbesartan compliance and urine collection times.
e. Patients took the IP twice daily for 16 weeks in Treatment Period 1 and Treatment Period 2 according to the randomisation schedule
f. For these visits, patients were required to take their dose of IP at the study site (not at home)
g. The 24-hour urine collection required for Screening commenced post visit after consent was obtained.
h. Rescreening could have been permitted for patients if their Baseline PCR value was not within ± 30% of the Screening value.
i. The flagged assessments were performed, but these data were not captured within the study database and were analysed on a post-hoc basis following database lock.
j. Inflammatory biomarker panel evaluating IL-6, IL-8, transforming growth factor-b, CTGF and NGAL

Supplementary Table S3: Baseline characteristics at start of placebo or DMX-200 treatment periods

Variable (Unit)	Placebo (N=8)	DMX-200 (N=8)
Urine PCR (mg/mmol), mean (SD)	285.6 (90.4)	361.0 (215.7)
Urine ACR (mg/mmol), mean (SD)	279.5 (88.5)	341.4 (197.1)
Mean (SD) eGFR (mL/min/1.73 m^2)	55.8 (34.6)	53.8 (33.4)
Mean (SD) serum potassium (mmol/L)	4.5 (0.54)	4.4 (0.64)
Mean (SD) systolic blood pressure (mm Hg)	127.2 (9.5)	131.5 (10.1)
Mean (SD) diastolic blood pressure (mm Hg)	79.8 (4.1)	79.2 (5.8)

Abbreviations: eGFR = estimated glomerular filtration rate; PCR = protein/creatinine ratio; DMX-200 = Repagermanium; SD = standard deviation.
Note: Baseline was the mean of values from Week −2 and Week −1 for placebo and DMX-200 treatment periods respectively.

Supplementary Table S4: Adverse events in study population

System Organ Class<br>Preferred Term	DMX-200 (N=8) Patients (%) Events	Placebo (N=8) Patients (%) Events	Unassigned* (N=8) Patients (%) Events
Patients reporting at least one AE	7 (87.5%) 18	6 (75.0%) 20	4 (50.0%) 7
Ear and labyrinth disorders	0 (0.0%) 0	1 (12.5%) 1	0 (0.0%) 0
Vertigo	0 (0.0%) 0	1 (12.5%) 1	0 (0.0%) 0
Gastrointestinal disorders	2 (25.0%) 4	3 (37.5%) 4	0 (0.0%) 0
Abdominal distension	0 (0.0%) 0	1 (12.5%) 1	0 (0.0%) 0
Constipation	1 (12.5%) 1	0 (0.0%) 0	0 (0.0%) 0
Diarrhoea	0 (0.0%) 0	1 (12.5%) 1	0 (0.0%) 0
Dyspepsia	0 (0.0%) 0	1 (12.5%) 1	0 (0.0%) 0
Gastritis	1 (12.5%) 1	0 (0.0%) 0	0 (0.0%) 0
Large intestine polyp	1 (12.5%) 1	0 (0.0%) 0	0 (0.0%) 0
Nausea	1 (12.5%) 1	1 (12.5%) 1	0 (0.0%) 0
General disorders and administration site conditions	2 (25.0%) 2	2 (25.0%) 2	0 (0.0%) 0
Chest pain	1 (12.5%) 1	0 (0.0%) 0	0 (0.0%) 0
Fatigue	0 (0.0%) 0	1 (12.5%) 1	0 (0.0%) 0
Peripheral swelling	1 (12.5%) 1	0 (0.0%) 0	0 (0.0%) 0
Pyrexia	0 (0.0%) 0	1 (12.5%) 1	0 (0.0%) 0
Immune system disorders	0 (0.0%) 0	0 (0.0%) 0	1 (12.5%) 1
Seasonal allergy	0 (0.0%) 0	0 (0.0%) 0	1 (12.5%) 1
Infections and infestations	2 (25.0%) 2	1 (12.5%) 2	3 (37.5%) 3
Folliculitis	1 (12.5%) 1	0 (0.0%) 0	0 (0.0%) 0
Nail infection	0 (0.0%) 0	1 (12.5%) 1	0 (0.0%) 0
Upper respiratory tract infection	1 (12.5%) 1	0 (0.0%) 0	3 (37.5%) 3
Urinary tract infection	0 (0.0%) 0	1 (12.5%) 1	0 (0.0%) 0
Injury, poisoning and procedural complications	0 (0.0%) 0	0 (0.0%) 0	1 (12.5%) 1
Contusion	0 (0.0%) 0	0 (0.0%) 0	1 (12.5%) 1
Metabolism and nutrition disorders	1 (12.5%) 1	3 (37.5%) 4	0 (0.0%) 0
Acidosis	1 (12.5%) 1	0 (0.0%) 0	0 (0.0%) 0
Decreased appetite	0 (0.0%) 0	2 (25.0%) 2	0 (0.0%) 0
Gout	0 (0.0%) 0	1 (12.5%) 2	0 (0.0%) 0
Musculoskeletal and connective tissue disorders	4 (50.0%) 4	1 (12.5%) 1	0 (0.0%) 0
Back pain	1 (12.5%) 1	0 (0.0%) 0	0 (0.0%) 0
Exostosis	0 (0.0%) 0	1 (12.5%) 1	0 (0.0%) 0
Neck pain	1 (12.5%) 1	0 (0.0%) 0	0 (0.0%) 0
Osteopenia	1 (12.5%) 1	0 (0.0%) 0	0 (0.0%) 0
Tendonitis	1 (12.5%) 1	0 (0.0%) 0	0 (0.0%) 0
Nervous system disorders	0 (0.0%) 0	2 (25.0%) 4	1 (12.5%) 1
Dizziness	0 (0.0%) 0	1 (12.5%) 1	0 (0.0%) 0
Headache	0 (0.0%) 0	1 (12.5%) 3	1 (12.5%) 1
Renal and urinary disorders	0 (0.0%) 0	0 (0.0%) 0	1 (12.5%) 1
Renal impairment	0 (0.0%) 0	0 (0.0%) 0	1 (12.5%) 1
Respiratory, thoracic and mediastinal disorders	2 (25.0%) 3	0 (0.0%) 0	0 (0.0%) 0
Oropharyngeal pain	1 (12.5%) 1	0 (0.0%) 0	0 (0.0%) 0
Productive cough	1 (12.5%) 1	0 (0.0%) 0	0 (0.0%) 0

System Organ Class<br>Preferred Term	DMX-200<br>(N=8)<br>Patients (%) Events	Placebo<br>(N=8)<br>Patients (%)<br>Events	Unassigned*<br>(N=8)<br>Patients (%)<br>Events
Rhinorrhoea	1 (12.5%) 1	0 (0.0%) 0	0 (0.0%) 0
Skin and subcutaneous tissue disorders	0 (0.0%) 0	1 (12.5%) 1	0 (0.0%) 0
Pruritus	0 (0.0%) 0	1 (12.5%) 1	0 (0.0%) 0
Vascular disorders	2 (25.0%) 2	1 (12.5%) 1	0 (0.0%) 0
Hypertension	2 (25.0%) 2	1 (12.5%) 1	0 (0.0%) 0

* Unassigned if (1) AE Start Date more than 28 days after last dose in Period 1 (and before the start dose in Period 2), OR
(2) AE Start Date more than 28 days after last dose in Period 2

Supplementary Table S5: Analysis of 24-Hour Urine Protein/Creatinine Ratio (UPCR) per treatment period and group.

**Urine PCR Period 1**		
	**Placebo (N=4)**	**DMX-200 (N=4)**
Baseline mg/mmol<br>Median (Range)	205.1 (196.9, 363.4)	374.6 (255.6, 798.9)
15/16 Weeks of Treatment mg/mmol<br>Median (Range)	216.8 (162.2, 489.5)	316.1 (151.4, 425.5)
Change from baseline mg/mmol<br>Median (Range)	11.8 (-34.7, 125.2)	-91.6 (-438.4, 27,05)
% Change from baseline<br>Median (Range)	5.7% (-17.6, 34.7)	-31.7% (-54.9, 6.8)
**Urine PCR Period 2**		
	**Placebo (N=4)**	**DMX-200 (N=4)**
Baseline mg/mmol<br>Median (Range)	341.4 (218.6, 413.1)	200.8 (157.4, 525.5)
15/16 Weeks of Treatment mg/mmol<br>Median (Range)	285.8 (115.3, 471.9)	209.2 (124, 462.7)
Change from baseline mg/mmol<br>Median (Range)	-22.2 (-186.3, 75)	-19.95 (-62.8, 23.1)
% Change from baseline<br>Median (Range)	-16.5% (-48.7, 25.0)	-3.4% (-27.9, 10.1)
**Urine PCR both periods**		
	**Placebo (N=8)**	**DMX-200 (N=8)**
Baseline mg/mmol<br>Median (Range)	259.5 (196.9, 413.0)	303.1 (157.5, 798.9)
15/16 Weeks of Treatment mg/mmol<br>Median (Range)	229.4 (115.3, 489.5)	262.2 (124, 462.7)
Change from baseline mg/mmol<br>Median (Range)	11.8 (-186.3, 126.2)	-55.4 (-438.4, 27.05)
% Change from baseline<br>Median (Range)	-1.0% (-48.7, 34.7)	-17.2% (-54.9, 10.1)

Supplementary Table S6: Analysis of Ratio of 24-Hour Urine Albumin/Creatinine Ratio (ACR)

	**Placebo (N=8)**	**DMX-200 (N=8)**
**Urine ACR**		
Baseline mg/mmol<br>Median (Range)	250.3 (179.1, 386.5)	297.8 (126.9, 707.8)
15/16 Weeks of Treatment mg/mmol<br>Median (Range)	218.3 (114.9, 463.5)	255.6 (117.9, 494.6)
Change from baseline mg/mmol<br>Median (Range)	1.85 (-230.6, 85.9)	-28.7 (-326.9, 21.8)
**Mixed-Model Analysis**		
Geometric mean (95% CI)	0.86 (0.62, 1.19)	0.82 (0.59, 1.14)
Placebo-corrected ratio<br>(DMX-200 versus placebo) (95% CI)		0.95 (0.65, 1.41)

Supplementary Figure S1: Schematic of the study design. B/L = Baseline (1 week); Follow = follow-up period (4 weeks after last dose of treatment); WO = washout (6 weeks).

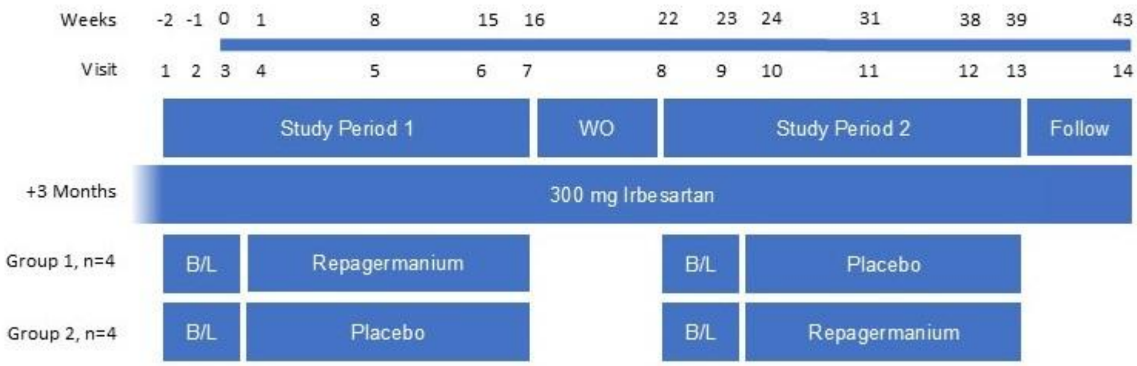

CONSORT 2010 checklist of information to include when reporting a randomised trial*

Section/Topic	Item No	Checklist item	Reported on page No
**Title and abstract**			
	1a	Identification as a randomised trial in the title	1
	1b	Structured summary of trial design, methods, results, and conclusions (for specific guidance see CONSORT for abstracts)	N/A
**Introduction**			
Background and objectives	2a	Scientific background and explanation of rationale	2-3
	2b	Specific objectives or hypotheses	3
**Methods**			
Trial design	3a	Description of trial design (such as parallel, factorial) including allocation ratio	Supplementary Material
	3b	Important changes to methods after trial commencement (such as eligibility criteria), with reasons	N/A
Participants	4a	Eligibility criteria for participants	Supplementary Material
	4b	Settings and locations where the data were collected	Supplementary Material
Interventions	5	The interventions for each group with sufficient details to allow replication, including how and when they were actually administered	Supplementary Material
Outcomes	6a	Completely defined pre-specified primary and secondary outcome measures, including how and when they were assessed	Supplementary Material
	6b	Any changes to trial outcomes after the trial commenced, with reasons	N/A
Sample size	7a	How sample size was determined	N/A
	7b	When applicable, explanation of any interim analyses and stopping guidelines	N/A
Randomisation:			
Sequence generation	8a	Method used to generate the random allocation sequence	Supplementary Material

	8b	Type of randomisation; details of any restriction (such as blocking and block size)	Supplementary Material
Allocation concealment mechanism	9	Mechanism used to implement the random allocation sequence (such as sequentially numbered containers), describing any steps taken to conceal the sequence until interventions were assigned	N/A
Implementation	10	Who generated the random allocation sequence, who enrolled participants, and who assigned participants to interventions	N/A
Blinding	11a	If done, who was blinded after assignment to interventions (for example, participants, care providers, those assessing outcomes) and how	N/A
	11b	If relevant, description of the similarity of interventions	N/A
Statistical methods	12a	Statistical methods used to compare groups for primary and secondary outcomes	Supplementary Material
	12b	Methods for additional analyses, such as subgroup analyses and adjusted analyses	Supplementary Material
**Results**			
Participant flow (a diagram is strongly recommended)	13a	For each group, the numbers of participants who were randomly assigned, received intended treatment, and were analysed for the primary outcome	Figure1
	13b	For each group, losses and exclusions after randomisation, together with reasons	Supplementary Material
Recruitment	14a	Dates defining the periods of recruitment and follow-up	Supplementary Material
	14b	Why the trial ended or was stopped	N/A
Baseline data	15	A table showing baseline demographic and clinical characteristics for each group	11
Numbers analysed	16	For each group, number of participants (denominator) included in each analysis and whether the analysis was by original assigned groups	4
Outcomes and estimation	17a	For each primary and secondary outcome, results for each group, and the estimated effect size and its precision (such as 95% confidence interval)	4-5
	17b	For binary outcomes, presentation of both absolute and relative effect sizes is recommended	N/A
Ancillary analyses	18	Results of any other analyses performed, including subgroup analyses and adjusted analyses, distinguishing pre-specified from exploratory	4-5 Supplementary Material

Harms	19	All important harms or unintended effects in each group (for specific guidance see CONSORT for harms)	N/A
**Discussion**			
Limitations	20	Trial limitations, addressing sources of potential bias, imprecision, and, if relevant, multiplicity of analyses	6
Generalisability	21	Generalisability (external validity, applicability) of the trial findings	6-7
Interpretation	22	Interpretation consistent with results, balancing benefits and harms, and considering other relevant evidence	6-7
**Other information**			
Registration	23	Registration number and name of trial registry	Supplementary Material
Protocol	24	Where the full trial protocol can be accessed, if available	N/A
Funding	25	Sources of funding and other support (such as supply of drugs), role of funders	7-8

Citation: Schulz KF, Altman DG, Moher D, for the CONSORT Group. CONSORT 2010 Statement: updated guidelines for reporting parallel group randomised trials. BMC Medicine. 2010;8:18.
© 2010 Schulz et al. This is an Open Access article distributed under the terms of the Creative Commons Attribution License (http://creativecommons.org/licenses/by/2.0), which permits unrestricted use, distribution, and reproduction in any medium, provided the original work is properly cited.

*We strongly recommend reading this statement in conjunction with the CONSORT 2010 Explanation and Elaboration for important clarifications on all the items. If relevant, we also recommend reading CONSORT extensions for cluster randomised trials, non-inferiority and equivalence trials, non-pharmacological treatments, herbal interventions, and pragmatic trials. Additional extensions are forthcoming: for those and for up-to-date references relevant to this checklist, see www.consort-statement.org.
